# Over-the-Counter Monocyclic Non-Steroidal Anti-Inflammatory Drugs in Environment—Sources, Risks, Biodegradation

**DOI:** 10.1007/s11270-015-2622-0

**Published:** 2015-09-30

**Authors:** Ariel Marchlewicz, Urszula Guzik, Danuta Wojcieszyńska

**Affiliations:** Department of Biochemistry, Faculty of Biology and Environmental Protection, University of Silesia in Katowice, Jagiellonska 28, 40-032 Katowice, Poland

**Keywords:** Monocyclic non-steroidal anti-inflammatory drugs, Toxicity, Biodegradation, Microorganisms

## Abstract

Recently, the increased use of monocyclic non-steroidal anti-inflammatory drugs has resulted in their presence in the environment. This may have potential negative effects on living organisms. The biotransformation mechanisms of monocyclic non-steroidal anti-inflammatory drugs in the human body and in other mammals occur by hydroxylation and conjugation with glycine or glucuronic acid. Biotransformation/biodegradation of monocyclic non-steroidal anti-inflammatory drugs in the environment may be caused by fungal or bacterial microorganisms. Salicylic acid derivatives are degraded by catechol or gentisate as intermediates which are cleaved by dioxygenases. The key intermediate of the paracetamol degradation pathways is hydroquinone. Sometimes, after hydrolysis of this drug, 4-aminophenol is formed, which is a dead-end metabolite. Ibuprofen is metabolized by hydroxylation or activation with CoA, resulting in the formation of isobutylocatechol. The aim of this work is to attempt to summarize the knowledge about environmental risk connected with the presence of over-the-counter anti-inflammatory drugs, their sources and the biotransformation and/or biodegradation pathways of these drugs.

## Introduction

In an age of high level care of human health, many pharmaceuticals are commonly used to cure or prevent diseases and other ailments, such as headache, muscle pain, or inflammatory conditions. Presently, over-the-counter drugs are very popular, especially over-the-counter monocyclic and polycyclic non-steroidal anti-inflammatory drugs (NSAIDs). Among these drugs, the most popular and the most often used are monocyclic NSAIDs, such as ibuprofen, acetaminophen, and salicylic acid (and its derivatives, like mesalazine), due to their availability (Ziylan and Ince 2011). For example, the yearly intake of ibuprofen is up to 300 t in Germany, 162 t in England, and 58 t in Poland (Sosnowska et al. 2009; Guzik et al. 2013a). In the USA during 2001–2005, about 29 billion doses of paracetamol in all forms were sold (Li et al. 2014). The high intake of these widely available drugs may lead to their or their metabolites’ presence in the environment. In connection with the presence of NSAIDs in the environment, there is a risk of long-term exposure, causing chronic toxic effect in organisms living there. This may cause negative effects for living creatures and the accumulation of drugs or their metabolites in the food chain (Carlsson et al. 2006; Sosnowska et al. 2009). Current knowledge about the microbial metabolism of non-steroidal anti-inflammatory drugs is still very little, and the fact that we can find them in the environment suggests that sewage treatment plants are not currently adapted to completely remove these drugs before they reach the environment. These drugs and their metabolites are found in wastewater influent and effluent from wastewater treatment plants. For example, in Germany, acetylsalicylic acid was detected in the sewage effluents at 0.22 μg/L (Heberer 2002) and paracetamol was detected in groundwater used as a source of public drinking water in California at 1.89 μg/L (Li et al. 2014). Observed metabolites are formed as a result of the metabolism by activated sludge microorganisms or enter the treatment plants as the human body biotransformation products with municipal wastewater (Buser et al. 1999; Zwiener et al. 2002; Marco-Urrea et al. 2009; Ziylan and Ince 2011). The fate of medicines, including monocyclic NSAIDs, in the natural environmental is still less known. The main aim of this work is a compilation of the actual knowledge about sources and risks connected with the presence of monocyclic non-steroidal anti-inflammatory drugs in the environment. Moreover, authors describe microbiological degradation of the three most widespread painkillers, antipyretic and anti-inflammatory and over-the-counter drugs—acetylsalicylic acid, paracetamol, and ibuprofen.

## Sources of Pharmaceuticals in the Environment

The development of modern analytical methods makes it possible to detect NSAIDs in the environment. Considering the high intake of drugs, it may be assumed that this has an impact on the presence of pharmaceuticals in wastewaters and surface waters. The main sources of drugs that reach the environment are excreted in non-metabolized form or slightly modified, i.e., hydroxylated, conjugated, and disposed of through the toilet (Buser et al. 1999; Heberer 2002; Khan and Ongerth 2002; Zwiener et al. 2002; Metcalfe et al. 2004). Nearly half of respondents declared the disposal of medications in the household trash. That is why the presence of pharmaceuticals is expected in the landfill leachate or leachate-contaminated groundwater (Kuspis and Krenzelok 1996; Musson and Townsend 2009). Hospitals’ wastewaters and discharges from pharmaceutical production also constitute a significant source of pharmaceuticals in the environment. In many countries, hospitals and pharmaceutical factories do not have separate wastewater treatment plants (WWTP); therefore, these contaminants pass into the general wastewater treatment system (Metcalfe et al. 2004). The burdened sewage flows into WWTPs, but not all NSAIDs are removed in biological sewage treatment with activated sludge. Drug detection in WWTP effluent confirmed inadequacy of wastewater treatment plants to completely removing these pollutants from sewage (Ternes 1998; Buser et al. 1999; Tixier et al. 2003; Lee et al. 2005; Gómez et al. 2007; Salgado et al. 2010). Consequently, this leads to the detection of drugs even in surface waters such as lakes or rivers (Winkler et al. 2001; Dębska et al. 2005; Roberts and Thomas 2006; Vieno et al. 2006; Togola and Budzinski 2008; Pailler et al. 2009). Additionally, Kolpin et al. (2004) observed an increased concentration of drugs in downstream, above places of outflows from WWTP.

## Environmental Risk of Monocyclic Non-Steroidal Anti-Inflammatory Drugs

Although NSAIDs are observed in the environment in low concentrations, there is little known about the long-term effects of low concentrations of these drugs on living organisms. The most data about toxicity of ibuprofen, paracetamol, and acetylsalicylic acid are based on acute toxicity and short-term chronic toxicity tests (Table 1). In acute toxicity tests, high concentrations of substances that may result in unrealistic effects are usually used. In many cases, metabolites of drugs are not taken into account in toxicity tests; therefore, it is difficult to evaluate the real risk of NSAIDs and their metabolites on the environment (Webb 2004). Marco-Urrea et al. (2009), using Microtox toxicity test with *Photobacterium phosphoreum* as a tested organism, proved that hydroxylated derivatives of ibuprofen (which was also found in sewage and surface waters) are more toxic than the original compound. Pomati et al. (2004) showed that even microgram per liter concentration of ibuprofen can influence the growth of aquatic phototrophs. For example, *Lemna minor* exhibited inhibition of growth after 7-day exposure to low concentration of ibuprofen. Under these conditions, the little effect on abscisic acid production was also observed (Pomati et al. 2004; Brausch et al. 2012; Murdoch and Hay 2013). High sensitivity to ibuprofen was also found for phytoplankton. Depending on tested organisms, EC_50_ value was between 1 and 315 mg/L after 72–120-h exposition to this drug (Brausch et al. 2012).

**Table 1:** Toxicity of selected monocyclic NSAIDs

Organism	Drug	Duration	Concentration (mg/L)	References
*Daphnia magna*	ASA	24 h EC_50_	1468	Lilius et al. (1994)
*Artemia salina*	ASA	24 h EC_50_	382	Calleja et al. (1994)
*Streptocephalus proboscideus*	ASA	24 h EC_50_	178	Calleja et al. (1994)
*Daphnia magna*	ASA	24 h EC_50_	168	Calleja et al. (1994)
*Brachionus calyciflorus*	ASA	24 h EC_50_	141	Calleja et al. (1994)
*Desmodesmus subspicatus*	ASA	24 h EC_50_	106.7	Cleuvers (2004)
*Daphnia magna*	ASA	24 h EC_50_	88.1	Cleuvers (2004)
*Daphnia magna*	Ibuprofen	48 h EC_50_	51.4	Han et al. (2010)
*Moina macrocopa*	Ibuprofen	48 h EC_50_	72.6	Han et al. (2010)
*Keletonema coststum*	Ibuprofen	96 h EC_50_	7.1	Halling-Sorensen et al. (1998)
*Daphnia magna*	Ibuprofen	48 h EC_50_	9.06	Halling-Sorensen et al. (1998)
*Lepomismacrochirus*	Ibuprofen	96 h EC_50_	173	Halling-Sorensen et al. (1998)
*Thamnocephalus platyurus*	Ibuprofen	24 h EC_50_	19.59	Kim et al. (2009)
*Oryzias latipes*	Ibuprofen	96 h EC_50_	>100	Kim et al. (2009)
*Hydra attenuata*	Ibuprofen	96 h LC_50_	22.36	Quinn et al.(2008)
*Hydra attenuata*	Ibuprofen	96 h EC_50_	1.65	Quinn et al. (2008)
*Daphnia magna*	Ibuprofen	24 h EC_50_	101.2	Cleuvers (2004)
*Desmodesmus subspicatus*	Ibuprofen	24 h EC_50_	343.2	Cleuvers (2004)
*Artemia salina*	Paracetamol	24 h EC_50_	577	Calleja et al.(1994)
*Daphnia magna*	Paracetamol	24 h EC_50_	55.5	Calleja et al. (1994)
*Brachionus calyciflorus*	Paracetamol	24 h EC_50_	5306	Calleja et al. (1994)
*Daphnia magna*	Paracetamol	24 h EC_50_	13	Kühn et al. (1989))
*Daphnia magna*	Paracetamol	48 h EC_50_	9.2	Kühn et al. (1989))
*Daphnia magna*	Paracetamol	24 h EC_50_	293	Henschel et al. (1997)
*Daphnia magna*	Paracetamol	48 h EC_50_	50	Henschel et al. (1997)
*Brachydanio rerio*	Paracetamol	48 h EC_50_	378	Henschel et al. (1997)
*Scenedesmus subspicatus*	Paracetamol	24 h EC_50_	134	Henschel et al. (1997)
*Daphnia magna*	SA	24 h EC_50_	230	Wang and Lay (1989)
*Daphnia magna*	SA	EC_50_	118	Henschel et al. (1997)
*Brachydanio rerio*	SA	48 h EC_50_	37	Henschel et al. (1997)
*Scenedesmus subspicatus*	SA	72 h EC_50_	>100	Henschel et al. (1997)

*SA* salicylic acid, *ASA* acetylsalicylic acid (aspirin), *ND* no data

After 14 days of exposure of *Daphnia magna* to ibuprofen at concentration of 20, 40, and 80 mg/L, significant effects in the total reproduction of daphnias were observed. Reproduction decreased with the increase of the drug concentration and totally stopped at 80 mg/L. Additionally, the time of first reproduction was delayed in a concentration of 40 mg/L. A low concentration of ibuprofen within the range of 1–100 ng/L caused a decrease in the activity of *Gammarus pulex*. This is very important information, because this concentration range of ibuprofen corresponds more to the concentration in the environment (de Lange et al. 2006). Pounds et al. (2008) showed some chronic effects of ibuprofen on mollusc *Planorbis carinatus* in the drug concentrations 0.41, 1.02, 2.43, and 5.36 mg/L. In the lowest dose, the authors did not observe snails laying eggs. A dose of ibuprofen at 5.36 mg/L caused an inhibition of egg hatching (Pounds et al. 2008). Han et al. (2010) also observed a delay in *Oryzias latipes* egg hatching after exposure to 0.1 μg/L of ibuprofen. After 120 days of ibuprofen exposure, the survival of fish was also significantly lower when compared to the control population (Han et al. 2010). The obtained results are significant, because the drug concentration used in the experiment is observed in the environment (Dębska et al. 2005; Pailler et al. 2009).

Wu et al. (2012) described *p*-aminophenol as a major metabolite of paracetamol metabolism in microbes. 4-Aminophenol is one of the most toxic phenols, which causes the kidney or the liver damage (Newton et al. 1982; Song and Chen 2001). Li et al. (2014) observed the appearance of N-acetyl-*p*-benzoquinone imine (NAPQI) during biodegradation of acetaminophen in soil. Additionally, the increased sorption of paracetamol in soil biosolids was observed, which may cause a decrease of acetaminophen mineralization. Simultaneously, it may affect the half-life of drugs in the environment (Li et al. 2014).

N-acetyl-*p*-benzoquinone imine is also one of the first phase metabolites of paracetamol detoxification in humans, excreted as a glutathione conjugate with urine (Tsikas et al. 2011; Li et al. 2014). It is defined as highly hepatotoxic (Bender and MacCrehan 2006; Hinson et al. 2010; Tsikas et al. 2011). Bender et al. (2004) suggest that NAPQI may be a potent inhibitor of human topoisomerase IIα.

Toxicological research conducted in the presence of high concentration of NSAIDs does not provide information about the influence of long-term exposure to low concentration of drugs. The answer to this question may be provided only by long-term research of many generations of aquatic organisms living in the presence of low drug concentration (Rzepa 2009).

## Acetylsalicylic Acid Biodegradation by Microorganisms

In humans and animals, acetylsalicylic acid is immediately hydrolyzed to salicylic acid which can be removed from the body unchanged or in the form of conjugates with glycine (as a salicyluric acid) or with glucuronic acid, or hydroxylated at the C-5 position of the ring to gentisate (de Gaetano et al. 1985; Ingelman-Sundberg et al. 1991; Paterson et al. 2008). Apart from excretion from human and animal organisms, salicylic acid is a widespread molecule in plants. It plays a role in several physiological processes, like stomatal closure, flower induction, heat production and, most of all, its main function is defense against pathogen attack (Verberne et al. 2000). If we take this into consideration, it is not strange that in nature, there are effective salicylate degradation mechanisms. Many bacterial strains, like *Micrococcus*, *Sphingomonas*, *Amycolatopsis*, *Streptomyces*, *Pseudomonas*, *Alcaligenes*, *Pseudoramibacter*, *Rhodococcus* (Chakrabarty 1972; Shamsuzzaman and Barnsley 1974; Haribabu et al. 1984; Grund et al. 1990; Grund et al. 1992; Civilini et al. 1999; Hintner et al. 2001; Ishiyama et al. 2004; Deveryshetty et al. 2007; Jouanneau et al. 2007; Silva et al. 2007; Lanfranconi et al. 2009) and fungi, like *Sclerotinia*, *Trichosporon*, *Aspergillus*, *Fusarium*, *Rhodotorula*, *Cryptococcus* (Anderson and Dagley 1980; Kuswandi and Roberts 1992; Middelhoven 1993; Iwasaki et al. 2009; Qi et al. 2012; Penn and Daniel 2013) are capable of degrading salicylate (Table 2) via a few catabolic pathways.

**Table 2:** Microorganisms degrading selected monocyclic NSAIDs

Strain	Drug	Concentration	References
*Pseudomonas putida* R1	SA	10 mM	Chakrabarty (1972)
*Acinetobacter lwoffii*	ASA	2.77 mM	Grant (1971)
*Amycolatopsis rugosa* DSM 43387	SA	ND	Grund et al. (1990)
*Amycolatopsis rugosa* DSM 43388	SA	ND	Grund et al. (1990)
*Streptomyces niger* DSM 40302	SA	ND	Grund et al. (1990)
*Streptomyces olivaceiscloticus* DSM 40595	SA	ND	Grund et al. (1990)
*Rhodococcus* sp. B4	SA	ND	Grund et al. (1992)
*Pseudaminobacter salicylatoxidans* B12	SA	5 mM	Hintner et al. (2001)
*Pseudomonas* sp.	SA	3.5 mM	Shamsuzzaman and Barnsley (1974)
*Pseudomonas aeruginosa* 2NR	SA	ND	Civilini et al. (1999)
*Streptomyces* sp. WA46	SA	7.24 mM (on agar plate)	Ishiyama et al. (2004)
*Rhodococcus ruber* IEGM 77	ASA	1.38–2.77 mM	Ivshina et al. (2006)
*Pseudomonas* sp. PPD	SA	ND	Deveryshetty et al. (2007)
*Alcaligenes* sp. PPH	SA	ND	Deveryshetty et al. (2007)
*Sphingomonas* sp. CHY-1	SA	0.5–1 mM	Jouanneau et al. (2007)
*Pseudomonas fluorescens* HK44	SA	0.18–1.45 mM	Silva et al. (2007)
*Trichosporon cutaneum*	SA	ND	Anderson and Dagley (1980)
*Aspergillus nidulans*	SA	10 mM	Kuswandi and Roberts (1992)
*Fusarium graminearum*	SA	0.1–20 mM	Qi et al. (2012)
*Sclerotinia sclerotiorum*	SA	1–10 mM	Penn and Daniel (2013)
*Trichosporon moniliiforme* WU-0401	SA	70 mM	Iwasaki et al. (2009)
Rhodococcus spp.	Paracetamol	1.65–3.31 mM	Ivshina et al. (2006)
*Delftia tsuruhatensis*	Paracetamol	0.007 mM	de Gusseme et al. (2011)
*Pseudomonas aeruginosa*	Paracetamol	0.007 mM	de Gusseme et al. (2011)
*Stenotrophomonas* sp. f1	Paracetamol	2.64 mM	Zhang et al. (2013)
*Pseudomonas* sp. f2	Paracetamol	16.54 mM	Zhang et al. (2013)
*Pseudomonas* sp. fg-2	Paracetamol	13.23 mM	Zhang et al. (2013)
*Penicillium* sp.	Paracetamol	0.66 mM	Hart and Orr (1975)
Filamentous fungi	Paracetamol	0.99 mM	Huang et al. (2006)
*Nocardia* sp. NRRL 5646	Ibuprofen	4.85 mM	Chen and Rosazza (1994)
*Sphingomonas* Ibu-2	Ibuprofen	2.42 mM	Murdoch and Hay (2005)
*Patulinobacter* sp. I11	Ibuprofen	0.002 mM	Almeida et al. (2013)
*Bjerkandera* sp. R1	Ibuprofen	0.0049–0.0097 mM	Rodarte-Morales et al. (2011)
*Bjerkandera adusta*	Ibuprofen	0.0049-0.0097 mM	Rodarte-Morales et al. (2011)
*Phanerochaete chrysosporium*	Ibuprofen	0.0049–0.0097 mM	Rodarte-Morales et al. (2011)
*Trametes versicolor*	Ibuprofen	0.097 mM	Marco-Urrea et al. (2009)
*Irpex lacteus*	Ibuprofen	0.097 mM	Marco-Urrea et al. (2009)
*Ganoderma lucidum*	Ibuprofen	0.097 mM	Marco-Urrea et al. (2009)
*Phanerochaete chrysosporium*	Ibuprofen	0.097 mM	Marco-Urrea et al. (2009)

*SA* salicylic acid, *ASA* acetylsalicylic acid (aspirin), *ND* no data

The strategy for degradation of aromatic structure comprises hydroxylation and cleavage of the aromatic ring. Hydroxylation into the dihydroxylated intermediates, the first step in the oxidative degradation of aromatic compounds, is catalyzed by oxygenases belonging to three groups: hydroxylating dioxygenases, activated-ring monooxygenases, or nonactivated-ring monooxygenases. As a result of hydroxylation, the key intermediates such as catechol, protocatechuic acid, hydroxyquinol, or gentisic acid are formed. These products are substrates for ring-cleaving dioxygenases. Salicylates are mainly transformed to catechol and gentisate, which are cleaved in the next step by dioxygenases from two groups—intradiol or extradiol (Guzik et al. 2013b; Guzik et al. 2014).

Two of the most important enzymes involved in salicylate decomposition are salicylate 1-hydroxylase and salicylate 5-hydroxylase (monooxygenases). Salicylate monooxygenases belong to one of the three groups of hydroxylating oxygenases—activated-ring monooxygenases (Wojcieszyńska et al. 2011). The general structure of these groups includes a three-component protein with separate functional units: reductase with a flavin cofactor, ferrodoxin with a Rieskie-type iron-sulfur cluster [2Fe-2S] and hexamer-built α_3_β_3_terminal oxygenase with [2Fe-2S] a cluster and one nonheme iron(II) per α subunit (Mason and Cammack 1992; Bertini et al. 1996). These catalytic proteins are able to insert one atom of molecular oxygen into the structure of the aromatic ring, simultaneously reducing the second oxygen atom to water. All salicylate hydroxylases need NADH to remain active. The oxidation of NADH is directly connected with FAD reduction (Katagiri et al. 1965; Sze and Dagley 1984; Grund et al. 1992; Suzuki et al. 1996; Fuenmayor et al. 1998; Balashova et al. 2001; Zhou et al. 2002; Jouanneau et al. 2007).

Chakrabarty (1972) and Deveryshetty et al. (2007) examined the ability of *Pseudomonas putida* R1 and *Alcaligenes* sp. PPH, respectively, to degrade salicylate. It was decomposed to catechol, the key intermediate, and further to 2-hydroxymuconic semialdehyde as a product of ring fission. Because of the significant activity of catechol 2,3-dioxygenase the authors concluded that *P. putida* R1 was capable of *meta* cleavage. On the other hand, decomposition of salicylate via catechol may also run via *ortho* cleavage with *cis*,*cis*-muconic acid as an aromatic ring cleavage product. Not only bacteria, such as *Amycolatopsis*, *Streptomyces*, or *Pseudomonas*, but also fungi, like *Fusarium*, *Rhodotorula*, *Trichosporon*, and *Sclerotinia* show that kind of catechol ring fission (Shamsuzzaman and Barnsley 1974; Anderson and Dagley 1980; Sze and Dagley 1984; Grund et al. 1990; Fuenmayor et al. 1998; Civilini et al. 1999; Ishiyama et al. 2004; Iwasaki et al. 2009; Qi et al. 2012; Penn and Daniel 2013).

Salicylate degradation may also lead via hydroxylation to gentisate. For example, this pathway was reported for *Rhodococcus* sp. B4 or *Streptomyces* sp. WA46 strains (Grund et al. 1992; Ishiyama et al. 2004). Enzymes engaged in this pathway need for their activity NADH, CoA and ATP as cofactors. Salicylate is converted to gentisate via salicylyl-CoA and gentisyl-CoA. In the first step, salicylyl-AMP ligase and probably salicylyl-CoA synthetase catalyze the formation of the thioester bond between salicylate and CoA and create salicylyl-CoA. The next step is the hydroxylation by salicylyl-CoA 5-hydroxylase with the formation of gentisyl-CoA. Hydrolysis to gentisate opens the way to ring cleavage by gentisate 1,2-dioxygenase to maleylpyruvate which leads to central metabolism. Civilini et al. (1999) showed the ability of *Pseudomonas aeruginosa* 2NR to convert salicylate into both intermediates, calechol, and gentisate.

Iwasaki et al. (2009) described a different model of decomposition of salicylate via catechol by yeast *Trichosporon moniliiforme* WU-0401. In their study, a non-oxidative way with phenol as an intermediate of salicylate degradation to catechol was presented. Salicylate was immediately transformed to phenol with, simultaneously, decarboxylation, but without hydroxylation. Before that, phenol was hydroxylated to catechol and further cleaved in the *ortho* position.

Most bacteria degrade salicylate via oxidative decarboxylation to catechol or via hydroxylation to gentisate. *Pseudaminobacter salicylatoxidans* B12 is capable of direct ring cleavage using NADH-independent salicylate 1,2-dioxygenase forming 2-oxohepta-3,5-dienedioic acid as an aliphatic product (Hintner et al. 2001).

## Microbial Degradation and Transformation of Paracetamol

**Fig. 1:**
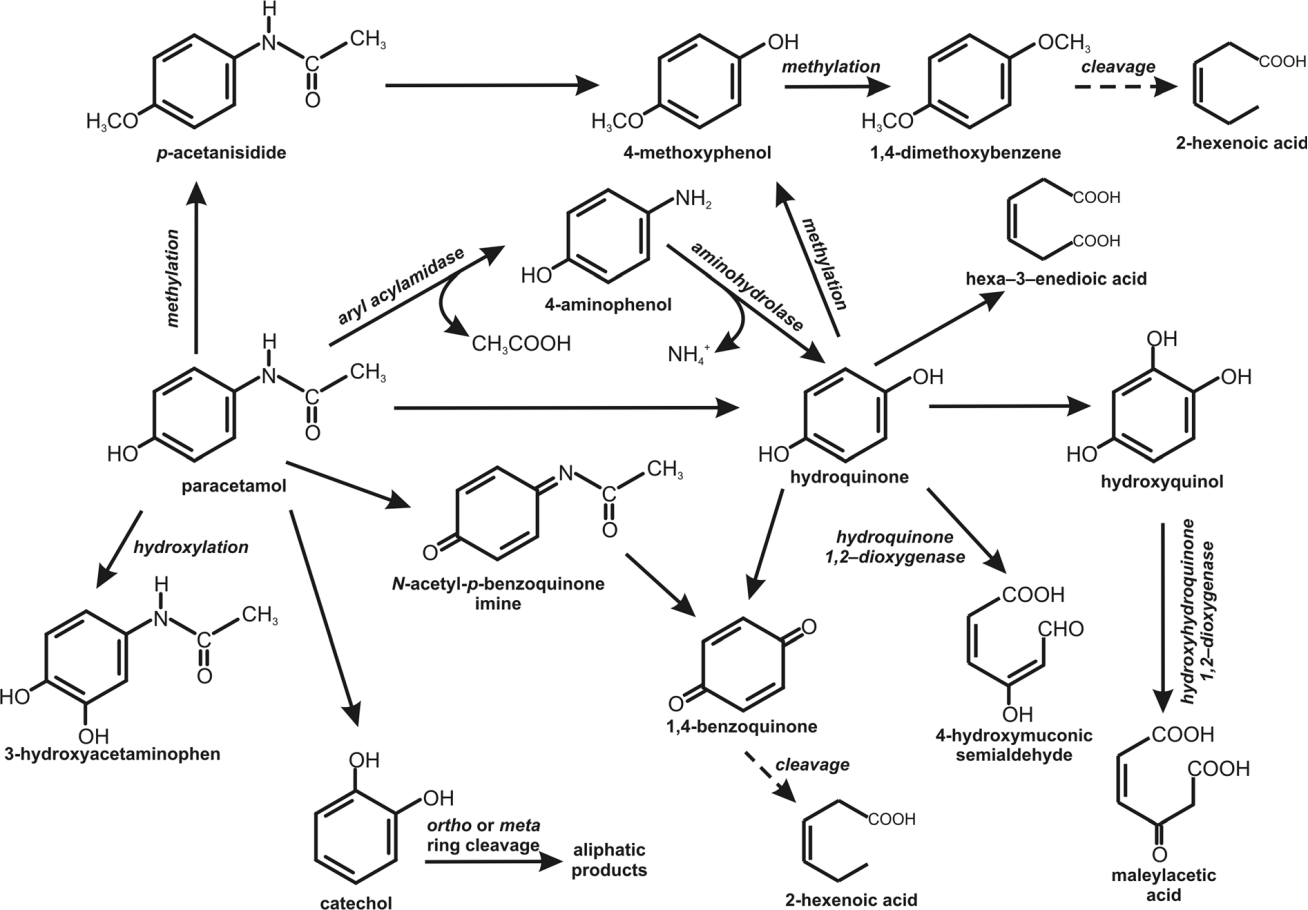
Biotransformation of paracetamol (Hart and Orr 1975; Takenaka et al. 2003; Kolvenbach et al. 2011; Wu et al. 2012; Zhang et al. 2013; Guzik et al. 2014; Li et al. 2014)

Paracetamol (acetaminophen) is an analgesic and antipyretic drug, and is one of the most popular over-the-counter drugs (Chandrasekharan et al. 2002). Acetaminophen is in most cases metabolized in the liver via three metabolic pathways. Most of this drug is secreted as conjugates with glucuronic acid (60 %) or sulfate (30 %) (Herd et al. 1991). A small amount of the medicine (8 %) may be dehydrogenated by cytochrome P450 to a toxic derivative N-acetyl-*p*-benzoquinone imine (Bock et al. 1987; Herd et al. 1991; Bessems and Vermeulen 2001). Nevertheless, knowledge about the further fate of acetaminophen in the environment is still limited. Hart and Orr (1975) obtained *Penicillium* sp. able to transform paracetamol to 4-aminophenol and acetate, probably with the use of aryl acylamidase. 4-Aminophenol is a dead-end metabolite (Fig. 1). Ivshina et al. (2006) reported the ability of *Rhodococcus* strains to degrade paracetamol with three detectable metabolites: 4-aminophenol, catechol, and hydroquinone. 4-Aminophenol may undergo oxidative deamination to hydroquinone (de Gusseme et al. 2011; Wei et al. 2011; Wu et al. 2012; Zhang et al. 2013). Further degradation of 1,4-hydroxybenzene could proceed in two ways. Hydroquinone may be directly cleaved by hydroquinone 1,2-dioxygenase with 4-hydroxymuconic semialdehyde as an aliphatic product (Jain et al. 1994; Rieble et al. 1994; Daubaras et al. 1996). This pathway was also suggested by de Gusseme et al. (2011), which observed degradation of hydroquinone by *Delftia tsuruhatensis* and *Pseudomonas aeruginosa*. The second way was described by Takenaka et al. (2003). These authors showed that *Burkholderia* sp. AK-4 converted 4-aminophenol to 1,4-hydroxybenzene and further to 1,2,4-trihydroxybenzene. Then 1,2,4-trihydroxybenzene was cleaved by hydroxyhydroquinone 1,2-dioxygenase to maleylacetic acid, which is introduced to the basic metabolism (Mason and Cammack 1992; Chauhan et al. 2000; Miyauchi et al. 1999; Moonen et al. 2008; Kolvenbach et al. 2011) (Fig. 1). Zhang et al. (2013) described the conversion of acetaminophen to hydroquinone, which was next transformed to an aliphatic product hexa-3-enedioic acid. Unfortunately, the authors did not determine the enzyme engaged in ring cleavage. However, it seems that hexa-3-enedioic acid was a product of aromatic ring fission or, if not, it that means some intermediate metabolites between aromatic and aliphatic compounds were passed over. Hexa-3-enedioic acid is similar to muconic acid—a product of *ortho ring* cleavage of catechol, but the authors did not find catechol in the studied samples. Based on reported intermediates a primary pathway of acetaminophen degradation could be proposed. The mechanism may be based on cutting off two carbon atoms in the form of formic acid (Fig. 1) (Zhang et al. 2013).

Furthermore, Huang et al. (2006) described the formation of glucoside conjugates with paracetamol by soil filamentous fungi via O- and N-linkages. This is a similar way to the human detoxication routes of xenobiotics in phase II of detoxication (Halling-Sorensen et al. 1998).

In 2014, Li et al. described degradation pathway of paracetamol in soil microorganisms. It was shown that in the first step, aromatic ring of paracetamol is hydroxylated to 3-hydroxyacetaminophen, oxygenated to N-acetyl-*p*-benzoquinone imine, or methylated to *p*-acetanisidide. It is suggested that cytochrome P-450 may be engaged in these processes. N-acetyl-*p*-benzoquinone imine is then metabolized to 1,4-benzoquinone which is more stable and critical toxic metabolite. *p*-Acetanisidide is transformed to 4-methoxyphenol and in the next step to the 1,4-dimethoxybenzene. The presence of 2-hexenoic acid in the soil extract suggests the cleavage of the aromatic ring of paracetamol (Li et al. 2014).

## Biodegradation/Biotransformation of Ibuprofen

Ibuprofen (2-(4-(2-methylpropyl)phenyl)propanoic acid) is one of the most popular and commonly used non-steroidal anti-inflammatory drugs. This makes it the third most popular drug in the world. Ibuprofen is also one of the dominating medicines present in sewage because of its relatively high therapeutic dose (600–1200 mg per day), and significant levels are excreted from the human body (even 70–80 %). This drug may be secreted as an unchanged molecule or as an unchanged molecule in conjugation with glucuronide (product of the second phase of detoxication that may be hydrolyzed in the environment) or as a few metabolites: hydroxyibuprofen (two isomers), carboxyibuprofen, and carboxyhydratropic acid (Halling-Sorensen et al. 1998; Buser et al. 1999; Zwiener et al. 2002). Nonetheless, little is still known about the environmental metabolism of ibuprofen, whose concentration in the environment ranges from nanograms per liter to micrograms per liter (Calamari et al. 2003; Bendz et al. 2005; Tauxe-Wuersch et al. 2005; Nakada et al. 2006; Roberts and Thomas 2006; Gómez et al. 2007; Lin et al. 2009; Pailler et al. 2009).

**Fig. 2:**
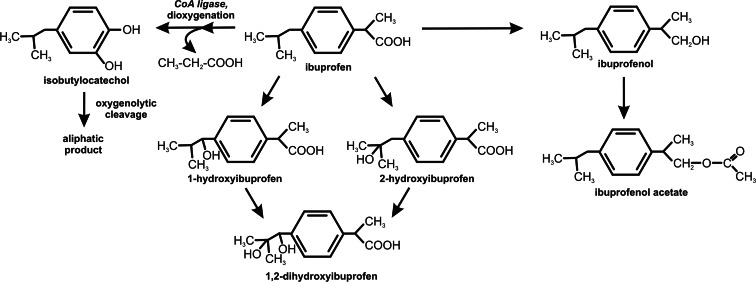
Microbiological transformation of ibuprofen (Chen and Rosazza 1994; Murdoch and Hay 2005; Quintana et al. 2005; Guzik et al. 2014)

Many reports describe only the initial steps of ibuprofen transformation. Rodarte-Morales et al. (2011) used three species of ligninolytic fungi: *Bjerkandera* sp. R1, *Bjerkandera adusta*, and *Phanerochaete chrysosporium* to check their ability to degrade pharmaceuticals, including ibuprofen (Table 2). They reported a rapid decrease of ibuprofen in growth medium, explaining that fact by degradation of the drug. However, these authors did not search for intermediates occurring in the degradation process, and they noted only the loss of the parent compound. It may be suggested that ibuprofen was not completely mineralized. *Trametes versicolor*, *Irpex lacteus*, *Ganoderma lucidum*, and *P. chrysosporium* are fungi capable of degrading ibuprofen (Marco-Urrea et al. 2009). All of them, excluding *P. chrysosporium*, degraded 10 mg/L ibuprofen to non-detectable levels. *P. chrysosporium* showed the lowest degradation level, between 70 and 88 %. Simultaneously, it should be noted that the authors tested the in vitro activity of laccase (also with laccase mediators) and manganese peroxidase. Moreover, they used inhibitors of the cytochrome P-450 complex to analyze the participation of these enzymes in ibuprofen degradation. In all cases, the researchers did not observe the contribution of the examined enzymes in the metabolism of ibuprofen. It may be suggested that the metabolism of ibuprofen could run through another pathway. The major metabolites which were found were hydroxylated in the isopropyl chain from ibuprofen—1- and 2-hydroxyibuprofen after the first hours of the experiment, and 1,2-dihydroxyibuprofen as a final metabolite after 7 days of cultivation (Fig. 2). Hydroxylated and carboxylated derivatives are frequent in the microbial metabolism (Zwiener et al. 2002; Quintana et al. 2005). It is noteworthy that ibuprofen derivatives are more toxic than the parent compound and may accumulate in the environment (Marco-Urrea et al. 2009). Despite low concentrations of these compounds in the ecosystem, they may be hazardous. However, the long-term effects of the organism exposure to this drug cannot be defined (Perry and Zylstra 2007). On the basis of European Union law, Cleuvers (2004) did not classify ibuprofen as toxic to aquatic organisms. However, a study with *Daphnia magna* and green algae showed that ibuprofen may be a toxic factor, especially in the presence of other drugs. This study informs about the effect of high-dose toxicity in short time exposure. In the environment, pharmaceuticals are not in high concentrations, so greater emphasis should be put on studies on the chronic toxicity of drugs (Cleuvers 2004).

During the degradation of ibuprofen by the lignolytic bacterium *Nocardia* sp. NRRL 5646, two metabolites, ibuprofenol and ibuprofenol acetate, were observed (Fig. 2). These products underwent further mineralization (Chen and Rosazza 1994).

Murdoch and Hay (2005, 2013) characterized one of the most completed ibuprofen degradation pathways in *Sphingomonas* Ibu-2 bacteria, capable of using ibuprofen as a source of carbon and energy. Based on genetic analyses, they proposed five-gene cluster (*ipfABDEF*), coding enzymes putatively involved in ibuprofen catabolism. Two of these genes (*ipfA*, *ipfB*) were very similar to genes coding two subunits of dioxygenases; the third gene was identified as a gene coding enzyme for the removal/addition of acyl groups—acyl-CoA synthetase (IpfD); the fourth one (*ipfF*) was described as a coenzyme A ligase gene; and for the gene *ipfE*, no function was found. Two additional genes *ipfH* and *ipfI* encode ferredoxin reductase and ferredoxin components of an aromatic dioxygenase system, respectively (Murdoch and Hay 2013). As the first step in the decomposition of ibuprofen by strain Ibu-2, thioesterification with coenzyme A with the participation of coenzyme A ligase was suggested. Removal of the propionic acid chain and dioxygenation reaction led to isobutylocatechol formation. This compound undergoes oxygenolytic cleavage (Murdoch and Hay 2005; Murdoch and Hay 2013) (Fig. 2). Ibuprofen biotransformation by *Variovorax* Ibu-1 occurs with trihydroxyibuprofen as a metabolite. This compound may be a dead-end metabolite or is substrate to the *meta*-ring fission reaction (Murdoch and Hay 2015).

*Patulinobacter* sp. I11 did not grow with ibuprofen as the only source of carbon and energy. However, degradation of ibuprofen was observed in the presence of yeast extract and tryptone. This suggests that ibuprofen could not induce the expression of enzymes responsible for its decomposition. In the bacterial genome, homologous genes were found coding enzymes potentially involved in ibuprofen decomposition, such as acyl-CoA synthetase, a protein containing a Rieske-like (2Fe-2S) iron-sulfur cluster (dioxygenase-like protein), and enoyl-CoA hydratase/isomerase (Almeida et al. 2013).

## Conclusion

The occurrence of micropollutants in the environment, such as non-steroidal anti-inflammatory drugs, is a relatively new problem. The presence of these drugs in the environment poses a risk of long-term exposure, causing chronic toxic effects for organisms. Although the salicylic acid pathway is very well described, little is known about the biotransformation/biodegradation of other non-steroidal anti-inflammatory drugs such as ibuprofen or paracetamol. Paracetamol degradation pathways lead through hydroquinone as a key intermediate, whereas ibuprofen is metabolized by hydroxylation or activation with CoA. However, sometimes biotransformation of monocyclic NSAIDs leads to the accumulation of intermediates more toxic than the parent compounds. An understanding the drug mineralization processes is key to creating commercially available solutions for this increasing problem.
